# The Therapeutic Effect of Artemisinin and Its Derivatives in Kidney Disease

**DOI:** 10.3389/fphar.2020.00380

**Published:** 2020-03-31

**Authors:** Ming Xia, Di Liu, Yu Liu, Hong Liu

**Affiliations:** Department of Nephrology, The Second Xiangya Hospital, Central South University, Hunan Key Laboratory of Kidney Disease and Blood Purification, Changsha, China

**Keywords:** artemisinin derivatives (artemisinins), kidney disease, inflammation, immunity, oxidative stress

## Abstract

Artemisinin (ARS) and its derivatives (ARSs) are recommended as the first-line antimalarial drugs for the treatment of malaria. Besides antimalarial function, its potent anti-inflammatory and immunoregulatory properties, as well as the ability to regulate oxidative stress have brought them to a prominent position. As researchers around the world are continually exploring the unknown biological activities of ARS derivatives, experimental studies have shown much progress in renal therapy. This review aims to give a brief overview of the current research on ARSs applications for kidney treatment with the evaluation of therapeutic properties and potential molecular mechanisms.

## Introduction

The imbalance between the molecular mechanisms that govern oxidative stress, inflammation, immunity, and cell death are important causes of acute kidney injury (AKI) and chronic kidney diseases (CKD) (Sureshbabu et al., 2015). Both AKI and CKD can lead to diminished kidney function and are associated with high mortality and morbidity. Accumulated evidence demonstrated that natural products are alternative sources for treating renal diseases on account of the conventional experience and multi-target characteristics (Chen et al., 2018).

**Figure 1:**
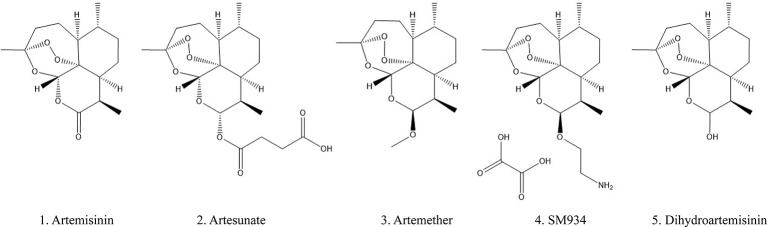
Chemical structures of artemisinin and its derivatives.

Artemisinin (ARS) is an effective constituent with a molecular weight of 282 originally extracted from traditional Chinese medicine *Artemisia annua L*, which was first discovered by Chinese scientists in 1972. Its chemical structure-sesquiterpene lactone with a peroxide bridge has been demonstrated to exert an excellent antimalarial effect (White et al., 2015; Chang, 2016). In the presence of heme or free iron, the production of reactive oxygen species and carbon-centered free radicals generated by the cleavage of the endoperoxide bridge can directly poison the parasites (Vennerstrom et al., 2004). ARS selectively kills plasmodium-infected red blood cells without destroying healthy cells, making it the recommended drug for the treatment of malaria (Lalloo et al., 2016) and more clinically effective than other antimalarial drugs such as hydroxychloroquine (HCQ) and chloroquine (CQ) (Golenser et al., 2006; Efferth and Kaina, 2010). ARS has a rapid onset of action and can be rapidly absorbed by the gastrointestinal tract after oral administration, with half-live ranging from 2 to 5 h. It is mainly distributed in the liver, kidney, and bile, and approximately 80% of the drug was excreted through the urine and feces within 24 h of administration (German and Aweeka, 2008; Li, 2012). Currently, a series of ARS derivatives (ARSs) with improved pharmacological features are used in clinical treatment including artemether (ARM), artesunate (ART), β-aminoarteether maleate (SM934), and dihydroartemisinin (DHA) (chemical structures were shown in **Figure 1**). The half-lives of ARM (2–4 h), ART (< 1 h), DHA (∼1 h) are shorter (Krishna et al., 2004; German and Aweeka, 2008) and oral intake represents a relatively safe route in the clinic.

In addition to decades of remarkable progress against malaria, studies have demonstrated a variety of other pharmacological effects beyond antimalarial, such as anti-virus, anti-neoplastic, anti-inflammation as well as immunosuppressive effects (An J. et al., 2017). The properties of ARSs have been intensively reviewed in systemic lupus erythematosus, rheumatoid, arthritis, multiple sclerosis, etc (Shi et al., 2015; Mu and Wang, 2018), while the treatment of kidney disease has not been summarized. This review will focus on the proposed therapeutic properties and mechanisms of ARSs in kidney disease, and discuss the potential application of ARSs as novel agents for future treatment.

## Search Strategy

Comprehensive literature searches for candidate studies were undertaken in two English and three Chinese biomedical databases from inception through February 2020. These databases included PubMed, Springer, Chinese National Knowledge Infrastructure, WanFang Med Online, and Chinese Biomedical Databases. Searches were limited to studies in English and Chinese. The following terms were used in the search: “artemisinin,” “artemisinins,” “artesunate,” “dihydroartemisinin,” “artemether,” “SM934,” “β-aminoarteether maleate,” “kidney,” “renal,” “nephro,” “nephritis,” nephropathy.”

## Overview of Researches on ARSs in Renal Disease

The effects of ARSs were mainly studied on animal models and cells, with two clinical studies targeting lupus nephritis (LN). In a randomized, 5-year follow-up clinical trial for LN (Lu, 2002), the treatment group (ARS 0.6 g/d and cordyceps 3–4 g/d) was reported to improve 24 h urine protein, creatinine clearance rate, level of C3, and was more effective than the control group (tripterygium wilfordii polyglycosides tablets 1 mg/kg, three times a day and/or baoshenkang tablets 150 mg, three times a day). A 2-month randomized trial had similar findings (Li et al., 2011), treatment with ART (50 mg, twice a day) was reported to improve systemic symptoms and reduce the immunological activity index than either before treatment or the control group with tripterygium wilfordii polyglycosides tablets (10 mg, three times a day and/or prednisone 0.5 mg/kg/d). However, these two studies were not blinded and placebo-controlled, which may result in information bias, and observations of larger samples are still lacking.

**Table 1** summarized the characteristics of animal studies with ARSs treatment, covering LN, adriamycin nephropathy (AN), subtotal nephrectomy (SNx), IgA nephropathy (IgAN), diabetic nephropathy (DN), AKI, unilateral ureteral obstruction (UUO), pristine or lipopolysaccharide (LPS)-induced nephritis, nephrotic syndrome (NS), and Heymann nephritis (HN). *In vitro* cell models and *in vivo* animal models investigations for ARSs efficacy on kidney disease involve various aspects including oxidative stress, inflammation action, and immune response, we will describe the effects of ARSs in sections below.

**Table 1:** Study characteristics of animal experiments in kidney disease.

Animal model	Drug and dose	Application mode	Targets	Reference
LN mice	ARS (150 mg/kg/d)	p.o. for 8 weeks	↓TNF-α, ↓IL-6 in serum; ↓NF-kB, ↓NF-kB p65, ↓TGF-β1 in renal tissue	Wu X. et al., 2010
LN mice	ARS (150 mg/kg/d) + prednisone (3.225 mg/kg/d)	p.o. for 8 weeks	↑GRα, ↓GRβ in PBMC; ↑P300/CBP in renal tissue	Wu X. L. et al., 2011
LN mice	ARS (5.55 mg/kg/d) + HCQ (16.6 mg/kg/d)	p.o. for 8 weeks	↓Anti-dsDNA, ↓ANA, ↓IgG, ↓IFN-γ, ↓TNF-α, ↑TGF-β1 in serum; ↑KLF15, ↓NF-κB in renal tissue	Liang et al., 2018
LN mice	ART (125 mg/kg/d)	p.o. for 16 weeks	↓Anti dsDNA, ↓ANA, ↓MCP-1 in serum; ↓VEGF in renal tissue	Jin et al., 2007; Jin et al., 2009;
LN mice	ART (50 mg/kg/d)	p.o. for 16 weeks	↓ICAM-1 in renal tissue	Wang et al., 2010;
LN mice	SM934 (10 mg/kg/d)	p.o. for 4 weeks	↓IL-2, ↓IL-17, ↓IFN-γ, ↓Anti-dsDNA IgG in serum; ↓STAT-1, ↓STAT 3, ↓STAT5, ↓CD3+B220+CD4-CD8- T cells, ↓Th1, ↓Th17, ↑Treg in splenocytes	Hou et al., 2011
LN mice	SM934 (2.5, 5, 10 mg/kg/d)	p.o. for 8 weeks	↓IL-6, ↓IL-10, ↓IL-17, ↓IL-21 in serum; ↑B cell, ↑germinal center B cells, ↓activated B cells, ↓plasma cells in splenocytes; ↓Blimp-1, ↑BCL-6, ↓TLR7/9 in renal tissue	Gui et al., 2019
LN mice	DHA (5, 25, 125 mg/kg/d)	p.o. for 10 d	↓NF-κB, ↓NF-κB p65 in renal tissue	Dong et al., 2003
LN mice	DHA (5, 25, 125 mg/kg/d)	p.o. for 10 d	↓TNF-α in serum; ↓NF-κB, ↓NF-κB p65, ↑IκB-α in renal tissue	Li et al., 2006
LN mice	DHA (60 mg/kg/d); DHA (60 mg/kg/d) + Prednisolone (9 mg/kg/d)	p.o. for 8 weeks	↓Fractalkine, ↓NF-κB, ↓NF-κB p65 in renal tissue	You et al., 2014
LN mice	DHA (25, 50, 100 mg/kg/d)	p.o. for 12 weeks	↑SIGIR, ↓TLR4/NF-κB in renal tissue	Huang et al., 2015
AKI mice	DHA (20, 40, 80 mg/kg)	p.o. for 10 d (pretreated)	↓MDA, ↑GSH, ↑SOD activity in renal tissue	An Y. et al., 2017
AKI mice	DHA (50 mg/kg/d)	p.o. for 1 d	↑Occludin, ↓TNF-α in renal tissue	Cheng et al., 2018
AKI mice	DHA (20 mg/kg/d)	p.o. for 3 d (pretreated)	↓Apaf-1, ↓cleaved-caspase-3, ↓IL-1β, ↓IL-5, ↓IL-6, ↓IL-17A, ↓IFN-γ, ↓TNF-α, ↓CXCL1, ↓MCP-1, ↓MIP-2 in serum; ↓NF-κBp65, ↓MDA, ↓NO, ↑GSH, ↑CAT, ↑SOD activity in renal tissue	Liu et al., 2019
DN rats	ARS (300 mg/kg/d)	i.p. for 3, 6 weeks	↓PDGF-B, ↓TIMP-2, ↑MMP-2, ↓PKC activation in renal tissue	Zhang et al., 2014a; Zhang et al., 2014b; Zhang et al., 2014c
DN rats	ARS (300 mg/kg/d)	i.p. for 4 weeks	↓DNA binding activity of NF−κB, ↓c-fos, ↓c-jun, ↓DNA binding activity of AP-1 in renal tissue	Zhou et al., 2014a; Zhou et al., 2014b; Zhou et al., 2014c
DN rats	ARS (300 mg/kg/d)	p.o. for 4 weeks	Differentially gene expression profile	Xiang et al., 2019
DN rats	ART (10, 30 mg/kg/d)	p.o. for 12 weeks	↓TLR4, ↓IL-8 in renal tissue	Nie et al., 2015
DN rats	ARM (670 mg/kg/d)	p.o. for 12 weeks	↓H_2_O_2_, ↑PGC-1α in serum and urine; ↑mitochondrial MPC content in renal tissue	Han et al., 2019
IgAN rats	ARS (16.7 mg/kg/d) + HCQ (16.7 mg/kg/d); ARS (8.3 mg/kg/d) + HCQ (25 mg/kg/d)	p.o. for 90 d	↓Deposition of IgA immune complexes and C3 in renal tissue	Lin et al., 2016
IgAN rats	ARS (33.33 mg/kg/d); HCQ (33.33 mg/kg/d); AH (16.65 mg/kg/d, 33.33 mg/kg/d, 66.66 mg/kg/d, ARS: HCQ=1:3)	p.o. for 4 weeks	↓IL-4, ↓IL-17, ↑IFN-γ, ↓Th2, ↓Th17, ↑Th1, ↑Treg proportion in peripheral blood and spleen; ↓deposition of IgA immune complexes and C3 in renal tissue	Bai et al., 2019
IgAN rats	ART (25, 50 mg/kg/d)	p.o. for 4 weeks	↓MCP-1 in renal tissue	Mi et al., 2009
IgAN rats	ART (25, 50 mg/kg/d)	p.o. for 4 weeks	↓IL-2, ↓IL-6 in serum	Ma et al., 2009
UUO mice	ART (25, 50 mg/kg/d)	p.o. for 3, 7, 14, 21 d	↓α-SMA, ↓CTGF in renal tissue	Mi et al., 2007
UUO mice	ART (25, 50 mg/kg/d)	p.o. for 3, 7 d	↓NF-κB p65, ↑IkB-α, ↑Smad7 in renal tissue	Ma et al., 2010
UUO mice	ART (15, 30, 60 mg/kg/d)	p.o. for 14 d	↓Fibronectin, ↓collagen I, ↓α-SMA, ↑E-cadherin, ↓USAG-1, ↑BMP-7 in renal tissue	Cao et al., 2016
UUO mice	DHA (40 mg/kg/d)	p.o. for 14 d	↓Collagen I, ↓collagen III, ↓Fibronectin, ↓TGF-β1, ↓PCNA, ↓α-SMA, ↓P13k/AKT in renal tissue	Zhang et al., 2019
NS rats	ART (5 mg/kg/d)	i.p. for 28 d	↓Triglyceride, ↑albumin in serum; ↓polymorphonuclear and mononuclear cells infiltration in renal tissue	Razavi et al., 2007
Nephritis mice	DHA (20 mg/kg/d)	i.p. for 48 h	↓TNF-α, ↓IL-6 in serum	Wu P. et al., 2011
Nephritis mice	ART (28.8 mg/kg/d)	p.o. for 6 weeks	↓TNF-α, ↓IL-6 in serum; ↓α-SMA, ↓TLR4, ↓MyD88, ↓NF-κB p65, ↓TGF-β1, ↓caspase-3 in renal tissue	Wan and Li, 2017
AN rats	ARS (150 mg/kg/d)	p.o. for 4 weeks	↑Nephrin, ↑podocin in renal tissue	Wu et al., 2014
HN rats	ARS (100 mg/kg/d)	p.o. for 4 weeks	↑Podocyte, ↑nephrin, ↑podocin in urine	Liu et al., 2017
HN rats	SM934 (12.5, 25 mg/kg/d)	p.o. for 28 d	↑Podocin, ↑nephrin, ↑desmin, ↓TGF-β1, ↓Smad2/3 phosphorylation, ↑Smad7, ↓EMT in renal tissue	Li et al., 2015
SNx rats	ARS (100 mg/kg/d)	p.o. for 16 weeks	↓TGF‐β1, ↓FSP1, ↓CTGF, ↓NLRP3 activation, ↓ASC, ↓NF‐κB signaling pathway in renal tissue	Wen et al., 2019

LN, lupus nephritis; AN, adriamycin nephropathy; SNx, subtotal nephrectomy; IgAN, IgA nephropathy; DN, diabetic nephropathy; AKI, acute kidney injury; UUO, unilateral ureteral obstruction; NS, nephrotic syndrome; HN, Heymann nephritis; ARS, artemisinin; DHA, dihydroartemisinin; ART, artesunate; ARM, artemether; HCQ, hydroxychloroquine; AH, artemisinin combined with hydroxychloroquine; TNF-α, tumor necrosis factor alpha; BAFF, B cell activating factor; GRα, glucocorticoid receptors alpha; GRβ, glucocorticoid receptors beta; IFN-γ, interferon-gamma; IκB-α, nuclear factor of kappa light polypeptide gene enhancer in B cells inhibitor alpha; Apaf-1, apoptotic protease activating factor-1; CXCL1, chemokine (C-X-C motif) ligand 1; IL-1β, interleukin-1 beta; IL-2, interleukin-2; IL-6, interleukin-6; IL-5, interleukin-5; IL-17A, interleukin-17A; KLF15, Krüppel-like factor 15; MCP-1, monocyte chemoattractant protein 1; MyD88, myeloid differentiation primary response 88; NF-κB, nuclear factor kappa-light-chain-enhancer of activated B cells; NF-κB p65, nuclear factor-κB protein 65; P300, EP300 or E1A binding protein p300; CBP, CREB binding protein, STAT-1,-3,-5, signal transducer and activator of transcription-1,-3,-5, TGF-β1, transforming growth factor beta 1; Th1, T helper 1 cells; Th2, T helper 2 cells; Th17, T helper 17 cells; Treg, T regulatory cell; TLR4, toll-like receptor 4; anti-dsDNA, anti-double-stranded DNA; ANA, antinuclear antibody; FSP1, fibroblast specific protein; CTGF, connective tissue growth factor; NLRP3, nod-like receptor protein 3; ASC, apoptosis-associated speck-like protein containing CARD; MIP-2, macrophage inflammatory protein-2; MDA, malonyldialdehyde; NO, nitric oxide; GSH, glutathione peroxidase; SOD, superoxide dismutase; CAT, catalase; PCNA, proliferating cell nuclear antigen; α-SMA, α-smooth muscle actin; PI3K/AKT, phosphatidylinositol-3-kinase/protein kinase B; USAG-1, uterine sensitization-associated gene-1; BMP-7, bone morphogenetic protein-7; PGC-1α, peroxisome proliferator-activated receptor γ coactivator 1α; MPC, mitochondrial pyruvate carrier; EMT, epithelial-mesenchymal trans; Smad-2,-3,-7, mothers against decapentaplegic homolog-2,3,7. significance of ↓ and ↑ are at the 0.05 level.

## Mechanism of Arss in Kidney

### Oxidative Stress Regulation of Artemisinins

Oxidative stress is an important mediator in the development and progression of CKD and AKI and its complications due to increased production of reactive oxygen species (ROS) and diminished antioxidant capacity (Ruiz et al., 2013). In the condition of a surplus of ROS, ARSs were reported to exhibit an antioxidant effect (Kim et al., 2014; Yang et al., 2018; Liu et al., 2019). In addition, according to the characteristics of ARSs, tumor cells are more vulnerable due to higher levels of iron (Robert et al., 2005) and are more susceptible to further ROS insults induced by ARSs (Hamacher-Brady et al., 2011; Efferth, 2017). Accordingly, ARSs have the potential to treat kidney cancer, and the emergence of new technologies such as ARS-based smart nanomedicine offers more possibilities (Luo et al., 2019).

#### In Vitro

Receptor-interacting protein kinase 1 (RIP1) is verified to modulate mitochondrial ROS production *via* excessive generation of mitochondrial superoxide and depletion of GSH (Zhou et al., 2017). ART was reported to induce ROS production and cell death in human renal carcinoma cells, while pretreatment with RIP1 inhibitor or knockdown of RIP1 reverted ART-induced cytotoxicity (Chauhan et al., 2017).

#### In Vivo

Pretreated DHA or ARS could ameliorate oxidative stress in AKI mice by restoring malonyl dialdehyde (MDA), nitric oxide (NO), glutathione peroxidase (GSH), catalase (CAT), and superoxide dismutase (SOD) activity in the kidney (An Y. et al., 2017; Liu et al., 2019). In addition, ARM was shown to reduce the serum H_2_O_2_ level and elevated renal cortical PGC-1α expression, but it did not exert obvious effects on CAT and SOD expression in the renal cortex in DN (Han et al., 2019). In normal rats without oxidative stress, orally taken artemether-lumefantrine (1.14/6.86 mg/kg/d, twice a day) or artesunate-amodiaquine (2.86/8.58 mg/kg/d, twice a day) for 7 d did not apparently alter renal antioxidant status compared with the control. Although there was no significant alteration in kidney, liver, lung, and brain weights, the artesunate-amodiaquine group showed cardiotoxicity (decreased heart weight by 27.2% compared with control) (Otuechere et al., 2012).

To date, ARSs could trigger cell death by inducing oxidative stress, and could also resist oxidation to reduce cell damage. Detailed understanding of the molecular mechanisms and the events by which ARSs regulate oxidative stress to control cellular processes in different cells remain to be explored.

### Anti-Inflammation Effect of ARSs

Inflammation plays a pivotal role in the pathophysiological processes of kidney diseases and associated with renal injury (Ernandez and Mayadas, 2016). The anti-inflammatory effects of ARS have been widely recognized, including repression of nuclear factor-κB (NF-κB), toll-like receptors (TLRs), signal transducer and activator of transcription (STAT), and phosphatidylinositol-3-kinase (PI3K)/protein kinase B (AKT) activity (Aldieri et al., 2003; Ho et al., 2014; Shi et al., 2015), which are key factors mediating immune-inflammatory response and are associated with kidney disease progression (Ruiz-Andres et al., 2016).

#### In Vitro

ART ameliorated high glucose-induced injury by suppressing TLR4/NF-κB/nod-like receptor protein 3 (NLRP3) inflammasome pathway in rat glomerular mesangial cell (Sun et al., 2018).

#### In Vivo 

For LN mice, it has been reported that treatment with ARSs could decrease interferon-gamma (IFN-γ), tumor necrosis factor-alpha (TNF-α), interleukin-6 (IL-6) in serum, and blocked intercellular adhesion molecule-1 (ICAM-1), fractalkine, NF-κB signaling pathway in renal (Dong et al., 2003; Li et al., 2006; Wang et al., 2010; Wu X. et al., 2010; You et al., 2014; Liang et al., 2018). A similar effect was observed in the nephritis and septic AKI models (Wu P. et al., 2011; Wan and Li, 2017; Liu et al., 2019). Also, ARSs were reported to inhibit disease progression *via* downregulating renal monocyte chemoattractant protein 1 (MCP-1) expression in LN, IgAN, and septic AKI models (Mi et al., 2009; Jin et al., 2009; Ma et al., 2009; Liu et al., 2019). In addition, ARSs were proved to alleviate the tubule-interstitial inflammation and fibrosis by inhibiting NF‐κB and mothers against decapentaplegic homolog (Smad) signaling pathway in SNx rats, Heymann nephritis rats and UUO models (Ma et al., 2010; Li et al., 2015; Wen et al., 2019). ARSs were also reported to reduce diabetic kidney damage by inhibiting TLR4, IL-8, and the DNA-binding activity of NF−κB in renal (Zhou et al., 2014a; Nie et al., 2015).

These data indicate that ARSs act as anti-inflammatory drugs at multiple components of inflammation signals and have a potential therapeutic effect on disease activity.

### Immunoregulatory Effect of ARSs

Under physiological conditions, the kidney contributes to immune homeostasis, assist in the removal of metabolic wastes and toxins, and maintain peripheral tolerance. The disruption of immune homeostasis an autoimmune response, such as the occurrence of LN and IgAN, resulting in the loss of renal function (Tecklenborg et al., 2018). T cells, B cells, and macrophages, as well as cytokines, are involved in immune regulation and are activated to varying degrees depending on disease pattern. The activated pathogenic cells are more likely to lead the breakdown of the peroxide bridge structure of ARS (Shi et al., 2015).

#### In Vitro

Chemokine ligand 2 (CCL2) and single immunoglobulin IL-1-related receptor (SIGIRR) are involved in the inflammatory pathogenesis of LN, DHA was reported to inhibit CCL2 secretion and increase SIGIRR expression and protect LPS-induced HK-2 inflammation (Huang et al., 2015).

#### In Vivo

ARS combined with prednisone was reported to increase the sensitivity of glucocorticoid compared to the group administrated glucocorticoid only in LN (Wu X.L et al., 2011), which may offer a possibility of alleviating the common side effects of existing glucocorticoids or immunosuppressants. SM934 was shown to protect LN mice by inhibiting both Th1 cells and Th17 cells responses (Hou et al., 2011) and reduce the number of activated B cells by inhibiting the expression of TLR7/9 (Gui et al., 2019). SIGIRR, as an inhibitor of TLR signal transduction, could be elevated by DHA. This might be a negative immune-modulating way for DHA to slow the progression of LN (Huang et al., 2015). In addition, ARS combined with HCQ was shown to improve IgAN rats immunity (Lin et al., 2016), possibly *via* inhibiting the differentiation of Th2 and Th17 cells while promoting Th1 and Treg cells differential (Bai et al., 2019).

All these studies suggest that ARS family drugs are able to perform immunosuppressive functions primarily through suppressing the activation of pathogenic immune cells and have a regulatory effect on autoimmune diseases.

### Other Effects

#### Anti-Fibrosis

Myofibroblasts can be differentiated by the epithelial-mesenchymal transformation (EMT) process, and are primarily responsible for excessive extracellular matrix production. TGF-β1, smooth muscle actin (α-SMA) and connective tissue growth factor (CTGF), metalloprotease (MMP), bone morphogenetic protein (BMP) are all considered to be major regulators of EMT and renal fibrosis (Liu et al., 2018). For UUO, both *in vitro* and *in vivo* study showed anti-fibrosis effect of ARSs related to the inhibition of EMT, fibroblast proliferation, and collagen synthesis (Zhang et al., 2017). And upregulating BMP-7 and downregulating BMP antagonist-uterine sensitization-associated gene-1 (USAG-1) (Cao et al., 2016), or mitigating CTGF, α-SMA (Mi et al., 2007), or PI3K/AKT pathway (Zhang et al., 2019) are all possible mechanisms.

#### Anti-Proliferation

Glomerular mesangial cell proliferation is a common pathological change of glomerular disease, effective control of mesangial cell proliferation is of great clinical significance. ARSs were reported to exert an inhibitory effect on the proliferation of rat mesangial cells (MA et al., 2007a; Ma et al., 2007b), possibly by inducing apoptosis and downregulating inflammatory cytokines TNF-α and IL-6 (Wang et al., 2016) or enhance caspase-3 activity (Wu X.L. et al., 2010). Our team recently demonstrated that DHA could inhibit the proliferation of aIgA1-induced human mesangial cells through the mTOR signaling pathway *in vitro* (Xia et al., 2020). In addition, ARSs were found to inhibit renal carcinoma cell proliferation by inhibiting the expression of fascin (Zhang et al., 2018), meanwhile inhibiting colony formation, migration, invasion, and tumorigenesis (Yu et al., 2019).

#### Regulate Glomerular Filtration

Glomerular permeability is regulated by the glomerular filtration barrier (GFB), which composed of glomerular endothelium, the glomerular basement membrane, and the podocyte layer. The dysfunction of intercellular adhesion and connection will result in the loss of the structural and functional integrity of GFB and the occurrence of proteinuria (Mehta and Malik, 2006). ART was proved to reduce glomerular permeability and improve proteinuria in LN mice by inhibiting vascular endothelial growth factor (VEGF) (Jin et al., 2007). Studies also showed that DHA ameliorated the hyperpermeability of GFB by inhibiting TNF-α and maintaining occludin expression (Cheng et al., 2018) or elevation of vascular endothelial (VE)-cadherin expression in endothelial cells (Li et al., 2018). In addition, ARS was observed to attenuate podocyte effacement and fusion *via* nephrin and podocin regulation in adriamycin-induced nephropathy (Wu et al., 2014), and reduce the shedding of podocyte and excretion of nephrin and podocin in Heymann nephritis (Liu et al., 2017).

#### Anti-Virus

ARS was shown to be effective in inhibiting polyomavirus BK replication in primary human kidney cells (Sharma et al., 2014).

#### Renal-Protective.

ART was reported to ameliorate proteinuria and suppress the progression of NS (Razavi et al., 2007). Studies also showed that ARS could relieve renal lesions in DN rats, through inhibiting platelet-derived growth factor-B (PDGF-B) expression (Zhang et al., 2014a), metalloproteinase tissue inhibitor-2 (TIMP-2) (Zhang et al., 2014b), spatiotemporal dynamics activation of protein kinase c (PKC) (Zhang et al., 2014c) and its downstream c-fos and c-jun (Zhou et al., 2014b), and their heterodimer activator protein (AP-1) (Zhou et al., 2014c). The results from the high-throughput sequence from DN rats treated with ARS may identifying promising targets for future treatment (Xiang et al., 2019). In addition, kidney function was found to be improved in cases of malarial nephropathy after treatment with ARSs (Ezzedine et al., 2007; Calitri et al., 2014; Gleeson et al., 2019).

## Interaction, Safety, and Side Effects

The toxicity of ARSs in cell culture, animals (mice, rats, rabbits, dogs, monkeys), and human clinical trials were well described (Efferth and Kaina, 2010). Large clinical studies and meta-analyses did not show serious side effects, despite mild and self-limited effects including mild nausea, vomiting, and diarrhea (Mssusa et al., 2016). Individual patients may appear transient transaminase elevated and mild rash. Non-hematological side effects include mild hepatitis, neurological, renal, cutaneous, and cardiac manifestations were uncommon (Roussel et al., 2017). Rare severe adverse events include prolongation of the QTc interval and cardiac arrhythmias (in LiverTox, 2012).

In addition, animal studies showed that artesunate can reduce glomerular filtration rate, increase renal blood flow, and has certain organ toxicity (Campos et al., 2001; Otuechere et al., 2012), while in a clinical study, liver function, kidney function, and routine blood tests remained normal in most patients treated with artesunate (von Hagens et al., 2017). A systematic review and meta-analysis showed that the use of ARS-based combination therapy in adults, children, and pregnant women in the 2nd or 3rd trimester was relatively safe (Kovacs et al., 2016). The drug interactions of ARSs are relatively unknown, more rigorous and comprehensive studies of interaction mechanisms are needed, as well as monitoring the safety of ARSs, especially concerning the genotoxicity and embryotoxicity (Amorim et al., 2013).

## Conclusion and Future Directions

Much knowledge has been gained about the antimalarial drugs in recent years, and more attention has been paid to ARSs application for renal damages. Many years of laboratory applications and research proved that ARSs have excellent anti-inflammatory and immunoregulatory functions. It is also a good regulator of the balance between oxidation and oxidation resistance. The regulation of the glomerular barrier highlights a unique aspect of the use of ARSs in kidney disease.

Despite accumulating evidence on the use of ARSs, the literature on its potential as a treatment for renal disease is still insufficient due to the lack of randomized controlled clinical trials. The additive effects of ARSs in combined administration with immunosuppressants and the structure-activity relationship need to be further clarified. Investigation of the improved properties of ARSs analogs also facilitates the discovery of novel drug targets for kidney disease (Santos et al., 2015; Zuma et al., 2016; de Lange et al., 2018).

## Author Contributions

MX provided direction, collected related literature, and drafted the manuscript. DL and YL made significant revisions to the manuscript and directed the review to be more focused. HL gave the final approval for the article to be published. All authors have read and approved the final manuscript.

## Funding

This work was supported by a research grant (81770714), (81470947), and (81570622) from the National Natural Science Foundation of China. It also funded by the postgraduate innovation project of Central South University in China (Project no.2018zzts920).

## Conflict of Interest

The authors declare that the research was conducted in the absence of any commercial or financial relationships that could be construed as a potential conflict of interest.
